# Potential to curb the environmental burdens of American beef consumption using a novel plant-based beef substitute

**DOI:** 10.1371/journal.pone.0189029

**Published:** 2017-12-06

**Authors:** Benjamin Goldstein, Rebekah Moses, Norman Sammons, Morten Birkved

**Affiliations:** 1 Department of Management Engineering, Quantitative Sustainability Assessment Division, Technical University of Denmark, Kongens Lyngby, Denmark; 2 Impossible Foods, Redwood City, CA United States of America; Leibniz-Institut fur Pflanzengenetik und Kulturpflanzenforschung Gatersleben, GERMANY

## Abstract

The food demands of the United States (US) impart significant environmental pressures. The high rate of consumption of beef has been shown to be the largest driver of food-borne greenhouse gas emissions, water use and land occupation in the US diet. The environmental benefits of substituting animal products with vegetal foods are well documented, but significant psychological barriers persist in reducing meat consumption. Here we use life cycle assessment to appraise the environmental performance of a novel vegetal protein source in the mean US diet where it replaces ground beef, and in vegetarian and vegan diets where it substitutes for legumes, tofu and other protein sources. We find that relative to the mean US diet, vegetarian and vegan diets significantly reduce per-capita food-borne greenhouse gas emission (32% and 67%, respectively), blue water use (70% and 75%, respectively) and land occupation (70% and 79%, respectively), primarily in the form of rangeland. The substitution of 10%, 25% and 50% of ground beef with plant-based burger (PBB) at the national scale results in substantial reductions in annual US dietary greenhouse gas emissions (4.55–45.42 Mt CO_2_ equivalents), water consumption (1.30–12.00 km^3^) and land occupation (22300–190100 km^2^). Despite PBB’s elevated environmental pressures compared to other vegetal protein sources, we demonstrate that minimal risk exists for the disservices of PBB substitution in non-meat diets to outweigh the benefits of ground-beef substitution in the omnivorous American diet. Demand for plant-based oils in PBB production has the potential to increase land use pressures in biodiversity hotspots, though these could be obviated through responsible land stewardship. Although the apparent environmental benefits of the PBB are contingent on actual uptake of the product, this study demonstrates the potential for non-traditional protein substitutes to play a role in a transition towards more sustainable consumption regimes in the US and potentially abroad.

## 1. Introduction

The food-related environmental footprint of the United States (US) is among the highest in the world per capita [1,2], driven largely by animal-sourced products [3–5]. Of all livestock products, beef is the most environmentally taxing, both in terms of total global impacts from the sector and normalized per unit mass [1,6–9]. Studies of the US diet have pinpointed beef as a main driver of greenhouse gas (GHG) emissions (enteric fermentation, deforestation) [3,10], water use (hydration and feed irrigation) [11] and land occupation (primarily rangeland) [11,12]. Although domestic consumption has waned in recent years, beef remains a staple of the American diet [13–15], representing a key opportunity to attenuate the environmental impacts of US food consumption.

Amended production practices provide some capacity for impact reduction, but fall short of the savings associated with reduced consumption of cattle-based products [16–18]. Environmental hotspots in the US beef supply chain include impacts on climate, biodiversity and water quality from feed crop production and grazing, in addition to direct GHG emissions from enteric fermentation and manure management. Cattle require more mass, protein and energy in feed then is produced in the final meat [6,11,17,19,20], but the environmental intensity of this could be lowered using potions of crops not suitable for humans as feed [21,22]. Enteric fermentation could be combated through higher feed qualities or manipulation of rumen [9,18,16]. Manure management practices, including methane capture, reduced storage time and waste-to-energy recovery, could all produce environmental dividends [16]. Lastly, grazing on non-competitive marginal lands at suitable time intervals could also enhance performance [18,16]. However, US beef production is already amongst the most efficient globally, employing advanced breeding techniques, high quality feed and concentrated feeding operations for 97% of the stock [23]. Thus, the largest potential efficiency gains in beef production are located outside the US [9,18,24]. Moreover, some of the abovementioned end-of-pipe solutions produce environmental trade-offs (e.g. using non-edible crop for feed generally reduces feed quality, triggering increased enteric fermentation).

Substituting beef for lower intensity products including vegetal-sourced foods could net dividends beyond those associated with optimizing the US beef production system [7,20], but this strategy hinges on consumer adoption of alternatives. The nutritional role of beef in the US diet could be performed by plant-sourced foods using 10% of the land while producing 4% of the GHGs [12]. More ambitious actions including shifting from standard US to vegetarian and vegan diets, effecting 30% and 50% reductions in dietary GHGs, respectively [3], and reduced land use [25]. Although the demand for beef in the US is elastic [12,13], behavioral hurdles exist in getting Americans to trade beef for beans, with meat consumption in general increasing [15]. Eating beef (and meat in general) is tied to a host of social, psychological and hedonic factors: taste, the perception that a meal requires meat, communal eating practices, dietary guidelines and advertising espousing meat as an essential part of a healthy diet, etc. [26–29]. Combating the significant edible food waste in the US could also play a role, although improvement potential for meat is low compared to other food groups [3].

Given this backdrop, to maximally reduce the environmental impacts of beef in the US diet, improved production techniques and dietary shifts should be complemented by other tools. One option is novel protein sources that more authentically mimic meat than existing vegetal foods (e.g. soy-based, mycoprotein or gluten products), including ‘cellular agriculture,’ yeast culture, bioprinting, scalable arthropod farming, and plant-based functional equivalents [30]. Early stage studies have found that these technologies could theoretically produce beef substitutes at a fraction of the environmental and resource costs of traditional beef, but do not represent market-ready commercial operations where the most realistic short- to mid-term environmental gains exist [30]. In the case of cellular agriculture, few studies address the full life cycle of inputs and downstream processing associated with cellular agriculture, and existing studies were performed on bench-scale in-vitro methods (the ‘$300,000 test-tube hamburger’)[31,32].

The portfolio of foods on the market that could realistically be regarded as a plant-based equivalent to beef is narrow. One such product is the plant-based burger (PBB) by Impossible Foods, which is a substitute designed to match the experience of cooking and consuming ground beef [30]. By fulfilling the same gustatory, culinary and nutritional functions as traditional beef, the PBB aims to lower the adoption barrier associated with the consumption of vegetal proteins in lieu of animal products. The primary ingredients of the PBB include texturized wheat protein (wheat TVP), coconut oil, and potato protein. To deliver the same sensory characteristics of animal-sourced beef, the company developed a modified yeast culture to produce “heme” (leghemoglobin), a naturally occurring protein in the root nodules of leguminous plants that functions as an analog for the myoglobin that gives beef its distinct flavor and cooking characteristics [33].

Compared to a typical US beef production system, PBB requires less than one quarter of the resources as modeled according to pilot scale production data collected in 2015 and refined in 2016 to account for supply chain changes [34]. As is the case with beef, land and water use associated with production of PBB is dominated by raw ingredients (agricultural products, maintenance of the yeast culture) rather than by production or formulation. While the majority of emissions within the beef supply chain are derived from cattle (raw materials), PBB emissions impacts are split between raw materials and production stages.

Aside two studies of in-vitro cultured meat production relying on estimates for production inputs [31,32], there exists no published environmental assessments using primary data from operations above bench-scale of novel protein sources. Moreover, the hypothetical impacts of novel protein substitution at the aggregate US level remain unknown. Here we look at the potential environmental and resource implications of substituting ground beef in the 2010 mean US diet (MUD), and plant-based proteins in the hypothetical vegetarian (VEG) and vegan (VGN) diets with PBB, at individual and national scales. Lastly, the potential for negative environmental trade-offs due to PBB adoption by VEG and VGN Americans are examined.

## 2. Materials and methods

Life cycle assessment (LCA) is a widely used method to quantify the environmental impacts of food production systems [1,3]. LCA focuses at the processes along a supply chain that deliver a service, accounting for material and energy inputs, and chemical emissions to the environment (herein, life cycle inventory or LCI), thus providing an appraisal of system-wide environmental impacts and resource draws [35,36]. We apply LCA to the US food supply chain, setting the system boundaries as the agricultural and processing stages, excluding the distribution (transport and packaging), preparation and disposal phases of the life cycle. These omissions underestimate environmental impacts and resource use [37,38], but given uncertainties surrounding relevant data for these stages and their typically marginal contribution to the outcomes of other food LCAs [10,39,40], the majority of life-cycle impacts should be captured here.

Three different archetypical American dietary patterns are modeled: the mean-US (MUD), vegetarian (VEG) and vegan (VGN) diets. The MUD is constructed from the 2010 USDA’s loss-adjusted-food-availability estimates of per capita consumption of ~250 food items in the US [41]. The VEG and VGN are built from the USDA’s 2010 dietary guidelines for vegetarian and vegan diets consuming 2000 kcal per day [42] (in line with measured adult vegetarian energy intake [43]), adapted to actual US consumption regimes using the 2010 loss-adjusted data. For instance, USDA guidelines suggest 1.5 cups of dark green vegetables per week for the VEG. Here the constituent dark green vegetables (e.g. broccoli, kale, spinach, etc.) were provided in the same ratios as found in the 2010 MUD. USDA data on food waste at the consumer and retail levels are also included so the diets represent the production volumes drawn by each diet to meet final ingestion. See S1 Table for a full breakdown of the components of the modeled diets.

The effects of substituting 10%, 25% and 50% of total protein in American diets are examined using MUD, VEG and VGN as baseline diets, with PBB as replacement protein. PBB is nutritionally similar to ground beef in most respects, besides lacking cholesterol and containing carbohydrates (see S2 Table for laboratory analytics) and is substituted on a 1:1 mass basis in the MUD. A nutritionally equivalent mass of PBB replaces the blend of protein foods in the VEG and VGN diets (see S1 Table for further information). Given uncertainties in the amount of total beef as ground-beef ingested by Americans, values of 30% (see S1 Text for estimation method) and 50% [44] were used to assess the upper and lower PBB market penetration.

GHG emissions, water use and land occupation are evaluated: all metrics to which LCA is widely applied and accepted, and relevant to the impacts of beef production in the context of net environmental burdens from the US diet. GHG emissions are assessed using the IPCC 2013 methodology to convert from atmospheric chemical emissions to the equivalent mass of carbon dioxide to affect the same degree of radiative forcing over a 100 year period (kg CO_2_e). Water use is calculated as ‘blue water’, the volume of surface or groundwater used and evaporated or incorporated into a product [45]. Lastly, agricultural land occupation is assessed as the physical area occupied in m^2^ arable land according to the Impact 2002+ method [46].

Hybrid-LCA methodology is employed here, whereby LCIs of on-farm resource use and chemical emissions are derived from studies of individual agricultural operations, while those for food processing (slaughterhouse operation, vegetable and fruit canning, etc.) are taken using a top down methods, based on national economic input-output accounts. Previous LCAs of on-farm operations are used to gather the production inputs and emissions data for foods, which were combined with inventories of individual inputs (fertilizers, fuels, irrigation, etc.) from the ecoinvent 3.2 database (www.ecoinvent.org) to build a complete LCI for that food. Ecoinvent 3.2 also contains complete LCIs for several relevant foods, which are adapted to US production conditions (e.g. US electricity and irrigation). The Carnegie Mellon 2002 US input-output database (www.eiolca.net), providing LCIs per dollar economic output for 428 economic sectors, is linked with US food production volumes to estimate average resource and emissions inventories per mass food produced in (e.g. per kilogram canned vegetables or fruit). Combining these two data streams provides a complete LCI for the agricultural and processing stages. S1 Text further details the LCA method employed here and the construction of the LCIs from the supporting literature.

LCI data for PBB production are from early-stage, low-volume (hundreds of kilos/day capacity) production scale of both heme and burger manufacturing for current bill of material. The PBB model relies on certain literature-derived assumptions to estimate commercial scale production (mainly associated with fermentation substrate and energy use) and the results of the PBB LCA reflect both known and projected bill of material and production processes specific to 2015–2016 LCA development period. Because the PBB product continues to evolve, these impacts are likely to change as formulations and processes continue to improve, and should be viewed as a snapshot of current production technology. To ensure validity the PBB life cycle inventory and subsequent analysis presented in this paper, the inventory and assessment were independently vetted by an external independent LCA consultant and again by Quantis US following inventory updates. Data management is done in the LCA software SimaPro 8.2.0.0.

## 3. Results

**Fig 1:**
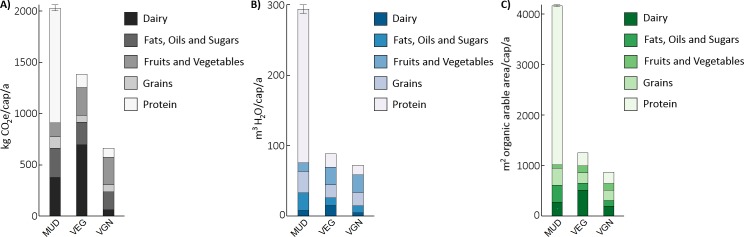
Results for the mean US Diet (MUD), vegetarian (VEG) and vegan (VGN). (A) GHG in kg CO_2_e. (B) Water use in m^3^ blue water consumption. (C) Land occupation in m^2^ organic arable land. Error bars indicate range of results for different proportions of ground beef in baseline MUD and varying carcass yields.

Fig 1 outlines the baseline results for the MUD, VEG and VGN for annual per capita GHG emissions (Fig 1A), water use (Fig 1B) and land occupation (Fig 1C). Error bars around the MUD represent different conversion rates from cattle live weight to beef (see S3 Table for detailed results). Diets are represented as the primary food groups comprising the USDA dietary recommendations as outlined in S1 Table. For instance, ‘Protein’ includes all foods whose primary functions are protein delivery, including eggs, meat, nuts, legumes, etc.

GHG emission results align with other US diet assessments, with shifts away from the MUD resulting in reduced GHG emissions for the VEG (-32%) and VGN (-67%). Of note is that if isocaloric diets were compared (total intake for MUD, VEG and VGN of 2481 kcal/day in line with 2010 USDA loss-adjusted numbers for the MUD), the reductions would have been -15% and -40% for the VEG and VGN, respectively. Protein dominates MUD impacts, with meat as the primary driver (50% total GHG emissions), itself impacted by beef (40–42% of total GHG emissions). The VEG is burdened by higher reliance on dairy as a protein and fat source, which elevate this dietary component’s impacts well above the MUD. Fruits and vegetables are the area of largest potential improvement for the VGN. Our findings are comparable to Heller et al.’s estimate of 5 kg CO_2_e/cap/d for the average American and reductions of 33% and 53% for vegetarian and vegan diets [3] and are in agreement with the scale of GHG emissions and reduction potentials through dietary shifts in nations with similar diets [37,47–49].

Water use follows the same pattern as GHGs: relative to the MUD, approximately 200 fewer cubic meters of water per annum are required to support the VEG (-70%) and VGN (-75%) though reductions shrunk when isocaloric diets were compared (-62% and -70% for VEG and VGN, respectively). The majority of the MUD’s impacts here stem from meat intake (74%), especially beef (56–58%), which requires sufficient animal hydration and significant embodied water inputs in feed via pasture, roughage, and concentrates. The VEG differs slightly from the VGN due to the former’s dairy and egg intake, but these differences are marginal when compared to the MUD. Eshel et al. [11] found that 150 m^3^/cap/a are needed for feed production for the US diet, in close alignment with our estimate of 140 m^3^/cap/a. Jalava et al. [50] also estimated significant reductions when moving from MUD to VGN, though their alternative method estimated larger savings of 438–657 m^3^/cap/a.

Significant decreases in land occupation also follow from a shift away from animal-based foodstuffs. The VEG and VGN occupy 70% and 79% less land than the MUD, respectively (VEG = -63% and VGN = -74% for isocaloric diet comparison). Of the MUD’s ~4100 m^2^ annual occupation, 75% is from meat, 67% from beef alone, where grazing land and feed production predominate. 92% of the land sparing is in the form of grazing for both dietary shifts, with the remainder consisting of high quality cropland. Much of this rangeland is poorly suited for crops, and hence, reductions in highly productive cropland are only a portion of total savings. Notwithstanding, land not recruited for cattle grazing or other forms of food production can serve as reservoirs of biodiversity, in addition to (and related with) performing the ecosystem services that support human life including: carbon sequestration, pollination, nutrient cycling, soil stabilization, flood mitigation, climate regulation [51]. Thus, switches to VEG and VGN could counteract the ecosystems degradation [52,53] and biodiversity loss [54] that have been noted in US grazing lands. Similar to GHG emissions, the VEG is greater than the VGN, exerted by dairy consumption and related agricultural space for feed crops. Our results match other LCAs of similar diets, where vegetarian and vegan diets effect 50–90% reductions in agricultural land occupation from omnivorous alternatives [37,47,49,55]. Of note is that inedible portions of plants can feed livestock to produce nutritionally dense animal products with limited environmental cost, and hence a diet with limited animal products could potentially have similar or lower land use to VEG and VGN diets contingent on the optimal balancing of residual resource and livestock production [22]. Moreover, exploring the use of agroecology principles in livestock production (enhanced genetic diversity, nutrient recycling, industrial symbiosis between farms, etc.) could also play a role in improving land use efficiency and quality on livestock farms [56], though these practices remain well outside the mainstream production practices in the US.

### 3.1 Beef substitution with PBB

**Fig 2:**
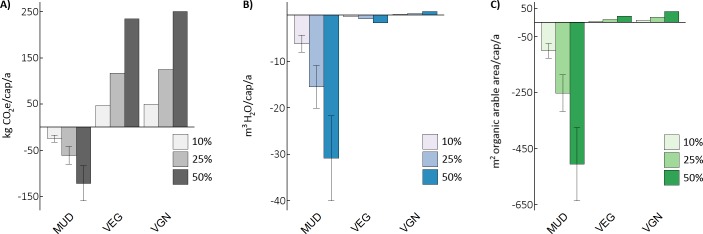
Per capita shifts in environmental burdens give PBB substitution in the mean US diet (MUD), vegetarian (VEG) and vegan (VGN). Substitution rates of 10%, 25% and 50% ground beef (MUD) and total protein foods (VEG and VGN). (A) GHG in kg CO_2_e. (B) Water use in m^3^ blue water consumption. (C) Land occupation in m^2^ organic arable land. Error bars indicate range of results for different proportions of ground beef in baseline MUD and varying carcass yields.

Fig 2 displays the potential impacts of PBB diffusion into the modeled diets at rates of 10%, 25% and 50%, where PBB substitutes for ground beef in the MUD, and a mix of vegetal proteins for the VEG and VGN. Upper and lower bounds of the MUD results represent high ground beef share of total beef intake (50%) combined with low conversion from live weight to beef (39%) and lower ground beef share of total beef (30%) combined with higher carcass yield (43%), respectively (see S1 Text for estimation methods).

By all three metrics the introduction of PBB improves the MUD’s environmental performance. Total dietary GHG emissions are reduced by 24 (1.2%), 61 (3.0%) and 122 (6.0%) kg CO_2_e/cap/a at increasing levels of diffusion. Of note is that a 50% PBB diffusion generates nearly half the GHG savings as an isocaloric switch to a vegetarian diet. Similarly, water use is reduced by 6 (2.1%), 15 (5.2%) and 31 (10.4%) m^3^/cap/a, while agricultural land occupation shrinks by 101 (2.4%), 252 (6.1%) and 505 (12.1%) m^2^/cap/a, primarily as spared rangeland. For the MUD, PBB provides an ecologically leaner protein option for GHGs (6.9 kg CO_2_e/kg PBB vs 30.1 kg CO_2_e/kg ground beef), water consumption (0.18 m^3^/kg PBB vs. 6.07 m^3^/kg ground beef) and land use (3.5 m^2^/kg PBB vs. 101.1 m^2^/kg ground beef). These reductions for the PBB are similar to those estimated for in-vitro meat production in GHGs (-75%) and land use (-94%) based on extrapolations from bench-scale data [31]. The results are more complex for the VEG and VGN diets. Notably increases are seen for GHG emissions (VEG: 3–17% and VGN: 8–38%), water impacts drop slightly for the VEG (0.4–1.8%) and rise for the VGN (0.2–1%), while cropland use increases marginally for both the VEG (0.3–1.7%) and VGN (0.9–4.4%). GHG emission increases stem largely from the energy inputs for the PBB, which are higher than soy and nut-based protein sources due to production processes and inclusion of leghemoglobin. Water and land remain essentially unaltered when moving from pulses, nuts and eggs to alternative plants sources, although the tendency for higher land occupation aligns with the lower protein content of wheat used in PBB compared to the fava beans used to model the legumes in the VEG and VGN.

The marginal shifts in dietary performance of the MUD at the individual level mask the true scope of reducing dietary environmental burdens from potential diffusion of such novel protein substitutes at the national scale. Considering the 299.40 million omnivores, 8.35 million vegetarians and 1.55 million vegans in the US [57], a hypothetical 10% introduction of PBB into all three diets would net annual reductions of 4.6–9.1 Mt CO_2_e GHG emissions, 1.3–2.4 Gm^3^ water use and 22300–38000 km^2^ agricultural land occupation. As above, most of the land use reduction is in the form of rangeland. To bring these numbers into context, this is the equivalent of removing 1.1–2.2 million cars from American roads annually (4400 kg CO_2_e/car/a [58]), eliminating the direct water consumption of 10.5–19.3 million Americans (124 m^3^/cap/a [59]) and freeing up an area equal to 1–1.6 times that of the state of New Hampshire. Table 1 highlights the potential impacts from PBB diffusions at higher levels.

**Table 1:** Net impacts of PBB at different substitution rates for protein in the MUD, VEG and VGN at US scale.

Indicator	% shift to PBB	Net Change	Analogue	Unit
GHGs	10%	4.55–9.08 Mt CO_2_e	1.13–2.25	million average US drivers removed
	25%	11.39–22.71 Mt CO_2_e	2.82–5.62	
	50%	22.78–45.42 Mt CO_2_e	5.64–11.24	
Water consumption	10%	1.30–2.40 km^3^	10.48–19.34	million fewer US water consumers
	25%	3.25–6.00 km^3^	26.20–48.37	
	50%	6.50–12.00 km^3^	52.41–96.74	
Land occupation	10%	22300–38000 km^2^	1–1.6	area of New Hampshire
	25%	55900–95100 km^2^	1–1.7	area of Illinois
	50%	111800–190100 km^2^	1–1.7	area of Nevada

## 4. Discussion

**Fig 3:**
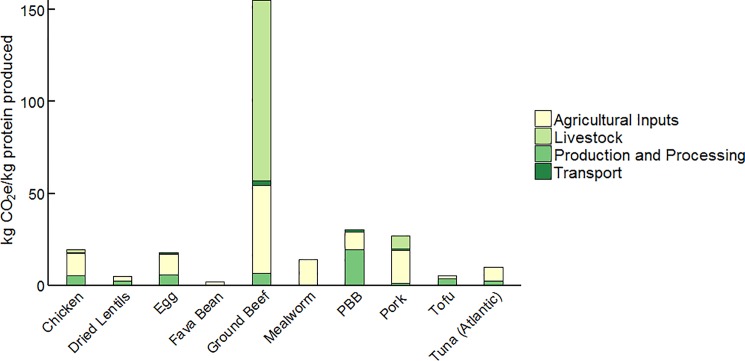
Embodied GHG for different foods. GHG emissions in kg CO2e/kg protein produced.

Dietary shifts from the MUD to the VEG or VGN, and substitution of PBB for ground beef, reduce food related pressures exerted from typical US residents. Actually achieving net gains is contingent on the adoption of PBB by a proportion of the 97% of US residents that are omnivores, since PBB uptake risks increasing some environmental pressures of the non-meat diets. Fig 3 compares GHG emissions for different common protein sources per kilogram protein delivered to the consumer’s plate. The PBB, though significantly lower in burdens than beef, is similar to other animal-sourced proteins and elevated above the other plant-based choices. It should be kept in mind that our GHG estimates for animal-sourced proteins could be considered conservative [60]. PBB appears more burdensome than protein from mealworms, though the numbers for the mealworm LCA are for a live product [61], excluding the processing inputs to convert live insects to more palatable end products.

**Fig 4:**
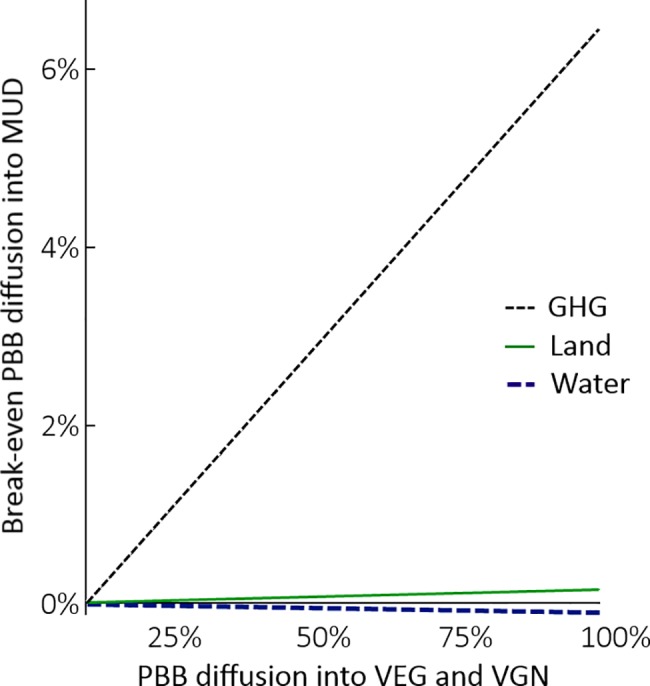
Required substitution rates of plant-based beef (PBB) in mean US diet (MUD) to counteract impacts from uptake by vegetarian and vegan Americans.

Notwithstanding, a small risk exists that increases in VEG and VGN environmental burdens for GHGs and land (and water for the VGN) from PBB adoption, might not be counteracted through uptake by the MUD. Fig 4 explores the required substitution of ground-beef with PBB in the MUD to balance 0–100% substitution of total protein with PBB in the VGN and VEG diets. In the extreme case that all vegetarians and vegans in the US source 100% of their protein from PBB, a replacement rate of around 6% ground beef (averaged ground beef as percentage of total beef and slaughtering efficiency) by PBB in the MUD would avoid a net increase, hinting that the potential risk for unintended increases of GHG emissions at the US aggregate is marginal. The same is true for land use, where a MUD penetration rate below 1% would suffice to counterbalance a net increase. For water consumption, the negative slopes indicate that the MUD would have to increase beef consumption to counteract net reductions of PBB uptake by the VEG and VGN; unlikely given falling US beef demand in recent decades [13].

Much of the beef reduction in the US diet has been matched by increases in poultry intake. Such a trend would be preferable from a GHG reduction perspective, even over PBB substitution or other novel meat substitutes [31,32]. However, the large scale industrialization of poultry since the 1970s has been undergirded by higher animal stocking densities and an undercutting of genetic diversity and resilience through a producer preference for fast-growing breeds [62], practices connected to the transmission of communicable diseases in the avian livestock population and high rates of antibiotic administration to industrial broilers to combat disease and hasten growth [62,63]. Similar practices have also been noted in US pork production [63,64]. Applications of antibiotics are linked to the increase of multi-drug resistant disease strains, diminishing the effectiveness of medicines in the human population [64–66]. Likewise, only considering GHG related impacts for tuna obscures the fact that shifting towards pescatarian habits would further stress marine ecosystems that have seen precipitous declines in population size, species richness and functional diversity at current fishing levels [67,68]. Such costs in other sustainability domains should be counted when comparing PBB to livestock products with potentially lower GHG emission intensities.

Large-scale sourcing for plant-based lipids could eventually present a land use change (LUC) risk, though that risk is dwarfed by the deforestation and LUC driven by beef production. Pasture and feedcrop expansion is the leading driver of forest loss and landscape intensification in Central and South America, [69–71] and one of several leading contributors to global net carbon losses (~880 MT C*yr^-1^) from wooded area conversion [72]. These types of land use change emissions have been successfully combated through legislation and through supply chain governance in the Brazilian Amazonia [73], but despite the progress of these efforts, these projects are not being continued in their current form [74]. Of concern is the sourcing of coconut oil for the PBB, as coconuts are grown in plantations in the humid tropics, regions that are rich in biodiversity and thus at elevated risk of habitat and species loss [54]. While coconut palm systems are of lower biodiversity value than intact forests [75], thus far, there has been limited recent detectable demand-driven extensification of coconut plantations in the source region, based on FAOSTAT land use and production volume data. Further, yield gaps in copra production indicate that production could be theoretically doubled without acreage expansion via cultivar selection and use of best management practices in coconut production (though such intensification is not without off-site environmental impacts) [76]. So while a net reduction in human appropriation of land in biodiversity hotspots seems probable when moving to PBB, oil sourcing will remain a potential sustainability challenge in the novel protein economy.

### 4. 1 Scaling up and future production efficiencies of PBB

Improvement potential exists for PBB, since significant impacts are borne in the processing of raw inputs to PBB, in addition to acquisition of raw materials. Future shifts from fossil fuel based electricity sources could net improvements in PBB GHG emission performance, since electricity accounts for 80% of emissions during the processing stage. Potential reductions in impacts from heme production appear likely as the technology matures and improved conversion efficiencies of raw inputs to heme are attained.

Taking the development of biofuels in recent decades as a barometer, considerable performance improvements are to be expected once PBB production reaches industrial scale using mature technologies. Precisely estimating the upscaling and technology maturation benefits and the resultant impacts on GHG performance of PBB production are difficult due to the novel nature of PBB and the scarcity of data on upscaling and maturation effects on environmental performance. Barlow et al. [77] showed that the net GHG emissions of algal derived biofuel improved from 80 g C02-eq/MJ to -44 g C02-eq/MJ as a result of scaling efficiencies of energy use (stirring, heating, etc.), elucidating the potential for improvement as biotechnologies move beyond pilot phase. Previous work on in-vitro meat production also assumed significant efficiencies with scaling and maturation [31,32], supporting the supposition that the environmental burdens of novel meat analogues such as a the PBB will likely decrease in the future.

Greening of the power needed by Impossible Foods may occur due a multitude of causes including changing the location of production to countries with more desirable grid mixes, construction of own (low GHG intensity) power supply and/or shifts of the regional US grid mix away from carbon intense fuels. The fossil fuel based energy mix used in this assessment accounts for a significant part of the climate change impacts produced by the PBB (see Fig 4). The global variation in the climate change intensity of one kWh ranges more than 2 orders of magnitude [78] meaning that choice of grid is paramount. For instance, the PBB’s electricity related impacts could be reduced by a factor 7–8 by producing on a grid with similar GHG intensity to France. LCAs of cultured meat production linked water use and GHG performance to production location, underscoring the importance of geographic specificity [32].

In contrast, significant improvements in the US livestock supply chain do not appear immediately forthcoming in most regards. The majority of beef GHG impacts stem from enteric fermentation, which is physiologically constrained, and though higher quality feeds do have the potential to mitigate a portion of these, North American beef production systems are already amongst the world’s leanest in this regard, limiting improvement potential through this route [9]. Although feed efficiency for US beef has increased since the mid 1970’s [79], long-term analysis of the US livestock production shows that feed to final product ratios have generally remained stable for all the staple livestock proteins throughout the 20^th^ century, with the exception of broilers which have seen substantial improvements [80]. The same can be said for current manure management practices [IBID]. Animal feed is also a major GHG source. Reduced tillage practices and improved yields would mitigate these, but given the advanced state of the majority of suppliers US production systems, such improvements are more salient to the low-tech livestock producers in the emerging economies [9,81]. Similarly, water use is physiologically constrained and strongly related to feed production. Exceptionally, land occupation could be significantly improved by switching from pasture to feedlot methods, though this potentially expands demand for arable cropland, and reduces demand for marginal rangeland.

### 4. 2 Additional aspects of US adoption of PBB

PBB adoption potentially reaps additional benefits not directly addressed through this assessment, including the reduction of reactive nitrogen runoff, a precursor to marine hypoxia and eutrophication. Livestock production is an important driver of these impacts at the regional and global from lax manure and urine management and runoff from fertilized feed crops [11,12,82]. PBB obviates both excrement production and the inefficiencies of converting feed to animal protein, ostensibly ameliorating eutrophication impacts in US beef supply regions, though more in depth analysis should buttress this claim.

Predicted increases in meat consumption at the global aggregate, particularly beef, will likely exacerbate stress on the planet’s bio-geochemical cycles. Production improvements [9] and proactive land stewardship [83] can mitigate these to an extent, but meeting future demand with PBB can complement these efforts to help move towards more sustainable diets in the US and elsewhere. In a globalized and interconnected world, the ability for US dietary trends to diffuse into other cultures is more pronounced than ever, including cultures ‘locked-in’ to similar consumption levels of beef (Europe, Australia) and those only now ramping up their beef demands (China, India, Africa, etc.). Capturing the latter regions is particularly important, since aggregate environmental impacts of beef consumption are expected to increase significantly, even when the substantial gains in beef production efficiency are included [1,9,18,24,80,84,85], and since large swathes of new agricultural land has recently been developed at the expense of tropical forests [86,87]. Moreover, as the US is currently a net exporter of beef [88], it is possible that US beef producers might simply export surplus production, hinting at the importance of dietary shifts beyond US borders.

It should be noted that contrary to the US and Latin America where ground beef is predominantly produced from dedicated beef herds, much of the ground beef in many European countries is a byproduct of spent dairy cattle and breeding overhead [89,90]. From an LCA perspective the GHG impacts of this type of beef are generally two thirds lower than those of a segregated beef herd due to co-product allocation [91,92], but still higher than those of PBB [90]. This relates again to the role of the Americas as a beef export region, since importing countries could be sourcing ground beef from more impacting locales, emphasizing the importance of consumption dynamics beyond the US border.

Essential to any discussion of the adoption of the PBB is the human factor. Changing diets is difficult and eating meat normalized in the United States [26–29]. Despite the PBB’s superior performance at the product level when compared to beef, estimates of aggregate changes from large-scale adoption are speculative. These benefits hinge on the uptake of the PBB, and the results at the country level only express the potential of novel protein sources to reduce environmental impacts at their current production efficiencies. Lastly, the PBB is one of numerous novel protein sources [30], each having a signature resource profile, meaning that the environmental outcome of their uptake at the national level is conjectural.

## 5. Conclusions

It has been long known that reducing meat intake, alongside improved production and management of food waste, can play an important role in reducing the environmental impacts of the US and similar diets. The challenge now is less about identifying the problem, but rather getting people to make a switch. This is a difficult proposition in the US where meat heavy diets are deeply enmeshed within its eating culture. Novel protein sources that substitute for environmentally deleterious livestock products while circumnavigating tough psychological hurdles offer a means to improve the environmental integrity of the MUD.

Novel protein substitutes, such as the PBB, could make important inroads to reducing the impacts of the MUD. When projected to the national level, the introduction of the PBB (and potentially other novel ground-beef replacements [31,32]) could generate substantial savings in GHG emissions, water consumption and agricultural land occupation. PBB has elevated net GHG emissions compared to other animal protein sources, but considering the age of the technology, it has substantial potential for improvement over animal-sourced foods, while providing benefits in additional realms of sustainability. PBB adoption can have slightly negative impacts on the VEG and VGN by some metrics, but a marginal uptake rate by the average American could counterbalance these.

## Supporting information

S1 Table Diets used in assessment. Table outlining the development of the baseline MUD, VEG and VGN diets, as well as their PBB substituted counterparts.
(XLSX)

S2 Table Nutritional data. Table outlining the nutritional properties of PBB and ground beef. Analytic methods for PBB are also noted.
(XLSX)

S3 Table Life cycle environmental impacts. Spreadsheet showing the life cycle environmental impacts for individual food items, diets on the per capita level and national level consumption for GHG emissions, water consumption and land occupation. Also includes changes on impacts from PBB diffusion at different rates.
(XLSX)

S1 Text Life cycle assessment methodology and life cycle inventories. PDF document detailing the life cycle assessment methodology employed here and the development of the life cycle inventories for the food products.
(DOCX)
