# Supplementary material for: Potential to curb the environmental burdens of American beef consumption using a novel plant-based beef substitute
Source: PLoS One. 2017 Dec 6;12(12):e0189029. doi: 10.1371/journal.pone.0189029 (PMC5718603; doi:10.1371/journal.pone.0189029)
Supplement: S1 Text — PDF document detailing the life cycle assessment methodology employed here and the development of the life cycle inventories for the food products. (DOCX) [file pone.0189029.s004.docx]

**S1 Text. Life cycle assessment methodology and life cycle inventories**

Supporting information for the article ‘Potential to curb the environmental burdens of American beef consumption using a novel plant based beef substitute’

1. **Overview:**

This study uses a hybrid life cycle assessment (LCA) approach that combines two different LCA methodologies: process-based LCA (P-LCA) and environmentally-extended-input-output LCA (EEIO-LCA). Figure 1 outlines the P-LCA methodology, which breaks down the life cycle of a product into different stages (material extraction, fabrication, distribution, etc.), accounts for the resource use and chemical emissions across all stages (aka. ‘life cycle inventory’), converts different emissions into a common unit for each impact category (e.g. CO_2_ equivalents for global warming) and then sums across the life cycle to estimate total environmental impacts. Here the scope of the assessment is on the agricultural production and processing stages as represented by the dashed line.

Resources from nature

Production of agricultural inputs

Farming

Food Processing

Packaging/ Distribution

Chemical emissions to environment

Preparation/ Consumption

Disposal

Here, all of the life cycle inventories (LCI) for agricultural production were built using P-LCA thinking, either with the ecoinvent 3.2 database or published results from previous LCAs. Data for food processing are not as readily available. EEIO-LCA was used to overcome this data gap.

EEIO-LCA works by augmenting standard economic input-output tables with environmental extensions outside of the economy. The foundation is **A,** the direct requirements matrix, representing interindustry (or sectoral) monetary exchanges. Each entry in the **A**, **a_i,j_**, represents the dollars demanded from industry **i** required by industry **j** to produce one dollar output from industry **j**. Vector **Y** represents demands from final consumers from each sector in dollars (i.e. excludes purchases from other industries that are used to produce goods for final consumption), where each element **y_i_** represents the total final demand from industry **i**. Vector **X** represents the total economic activity of both the final consumption and the interindustry exchanges. Thus the total demand, **X**, can be represented in matrix notation as [1]:

| [1] | $X=X\cdot A+Y$ |
| --- | --- |

Solving for total demand yields [2]:

| [2] | $X={(I-A)}^{-1}Y$ |
| --- | --- |

Where **I** is the identity matrix, and **(I-A)^-1^** is known as the Leontief inverse.

The equation is linear and scalable in that a change in final consumption **∆Y** can be related to a change in total production **∆X** in the form [3]:

| [3] | $\Delta X={(I-A)}^{-1}\Delta Y$ |
| --- | --- |

Environmental impacts can be included if emissions inventories from each industry (or sector) are known. For instance if total CO_2_ emissions are known for sector **i**, then the CO_2_ emissions per unit output, **R_i_**, can be determined as [4]:

| [4] | $R_{i}={total emissions}_{i}/X_{i}$ |
| --- | --- |

From [4] the emissions, **∆b**, due to an incremental change in final demand for sector can be taken as [5], where **R** is a vector of emissions factors per monetary unit output from each sector:

| [5] | $\Delta b={R(I-A)}^{-1}\Delta Y$ |
| --- | --- |

This type of environmental extension can be applied wherever inventories exist (land and water use, acidification, etc.) A wealth of resources exist for those further interested in EEIO-LCA ^1,2^.

Here EEIO data from the Carnegie Mellon IO database for the US economy were used to account for environmental impacts from food processing for US final consumption (www.eiolca.net). The Carnegie Mellon model accounts for 428 economic sectors in the US economy in the year 2002. Although the database is dated for some industries, seismic technological transitions have not occurred in US agricultural production (nor background energy systems) in the meantime, and so impacts should be generally representative of 2010 production. Moreover, using the database assumes that all foods imported from foreign economies are technologically equivalent to the US, an assumption that should hold given the US’s food security and low percentage of imports in the final consumption mix.

We only included the impacts from energy and chemical usage in food processing, since capital is typically of marginal contribution to total impacts. This is done by placing 0’s in the direct requirements matrix, A, for those supplying industries that are not related to energy supply or chemical production. Thus, for each food processing process, zeros are placed in all rows with the exception of the following industries:

- Oil and gas extraction
- Coal mining
- Other nonmetallic mineral mining and manufacturing
- Electric power generation, transmission and distribution
- Natural gas distribution
- Petroleum refineries
- Petroleum lubricating oil and grease manufacturing
- Industrial gas manufacturing
- Synthetic dye and pigment manufacturing
- Alkalies and chlorine manufacturing
- Carbon black manufacturing
- All other basic inorganic chemical manufacturing
- Other basic organic chemical manufacturing
- Biological product (except diagnostic) manufacturing
- Soap and cleaning compound manufacturing
- All other chemical product and preparation manufacturing

Because EEIO databases provide results in impacts per dollar final demand, it is necessary to convert this to impacts per unit mass so that they align with the mass-based P-LCA framework employed here. This was done using the USDA’s Loss Adjusted Food Availability (LAFA)^3^ data which allowed us to match the outputs from US sectors to the masses of food items produced in the baseline year. Table 1 shows the sectors used from the EEIO database, the dollars output from the sector^4^, the corresponding foods in each sector, the output of those foods for the baseline year from the LAFA data, and the dollars output per kilogram produced. As not all food items are available in the LAFA (e.g. fresh and frozen fish are lumped into a single value), the total mass output of each sector will be underestimated in some instances, inflating the conversion factor. However, the overestimations should be slight since the LAFA numbers include those foods consumed in the highest volumes by Americans. LAFA numbers also represent food produced for US consumption, excluding exports, which should also inflate some of the numbers. Thus, where possible USDA production statistics were used^5^, since these account for total US production of goods, including exports. This method generates the dollar demands for the average product produced by a sector, underestimating for some products and overestimating for others. Nonetheless it will provide a reasonable estimate of food processing related impacts.

| **EEIO Sector** | **Output (10^6^ 2002 USD)** | **Food Items** | **2002 Production (t)** | **Conversion (USD/kg)** |
| --- | --- | --- | --- | --- |
| Frozen Food Manufacturing | 33177 | All frozen food* | 21498825 | 1.54 |
| Fruit and vegetable canning, pickling, and drying | 39283 | Canned Fruit  Dried Fruit  Canned Vegetables  Dried Vegetables  Dried Beans | 2282605  1439323  13763065  4398280  945994  22829274 | 1.72 |
| Seafood product preparation and packaging | 4869 | Total Fish | 2140374 | 2.27 |
| Poultry processing | 45242 | Total Poultry** | 23554466 | 1.92 |
| Flour milling and malt manufacturing | 6030 | Total wheat flour | 18762937 | 0.32 |
| Wet corn milling | 1724 | Total corn products | 4071886 | 0.42 |
| Animal (except poultry) slaughtering, rendering, and processing | 89239 | Total red meat** | 22563360 | 4.15 |
| Total frozen dairy products | 4420 | Frozen Dairy | 3498392 | 1.26 |
| Dry, condensed, and evaporated dairy product manufacturing | 12948 | Evaporated/Condensed Milk | 6674302  508517  7182189 | 1.80 |
| Seasoning and dressing manufacturing | 16303 | Salads and cooking oils | 16303110 | 2.14 |

* LAFA data do not include data on highly-processed, ready-made meals, so total production volume taken as 2002 US total frozen food consumption ^6^

** Taken from USDA production reports^5^

With these conversion factors the P-LCA data can then be combined with EEIO-LCA data to generate LCIs encompassing both on-farm activities and upgrading from food processing. Figure 2 illustrates how the ecoinvent 3.2 P-LCA and Carnegie Mellon EEIO-LCA databases are combined to produce flour for the US market.

$0.32 ‘Flour milling and malt production’

Wheat production: modelled as ‘Wheat grain {US} | wheat production | Alloc Def, U’ from ecoinvent 3.2

Flour production process that combines EEIO and P-LCA databases

1 kg wheat grain

‘Flour milling and malt manufacturing’ from Carnegie Mellon database

1 kg ‘Wheat Flour {US}’

1. **Life cycle inventories:**
   1. **Ecoinvent based**

Ecoinvent 3.2 provides full LCIs for some food products, including processing. These were used where possible and adjusted for US production conditions when appropriate (e.g. changing the supplying electrical grid to US). Table 2 notes these processes, as they are named in S3, the ecoinvent 3.2 process that each one is based upon and any alterations to the original ecoinvent process (foods sourced primarily from the global market were not altered). Proceeding sections outline custom LCIs for food products for which no ecoinvent 3.2 surrogates could be found. Where multi-functional processes occurred, system expansion was attempted (e.g. producing system was credited for avoided production of by-products), but mass-based allocation was a performed for beef from culled dairy cattle and cashew nuts, since the former is unfairly biased by system expansion ^7^ and the latter had a plurality of by-products that are difficult to model with system expansion.

| **Process used here** | **ecoinvent 3.2 basis** | **Adaptions for study** |
| --- | --- | --- |
| Broccoli {US} | Broccoli {GLO}\| production \| Alloc Def, U | Electricity changed to ‘Electricity, low voltage {US}\| market group for \| Alloc Def, U’  Irrigation changed to ‘Irrigation {US}\| market for \| Alloc Def, U’ |
| Spinach {US} | Spinach {GLO}\| production \| Alloc Def, U | Electricity changed to ‘Electricity, low voltage {US}\| market group for \| Alloc Def, U’  Irrigation changed to ‘Irrigation {US}\| market for \| Alloc Def, U’ |
| Iceberg Lettuce {US} | Iceberg Lettuce {GLO}\| production \| Alloc Def, U | Electricity changed to ‘Electricity, low voltage {US}\| market group for \| Alloc Def, U’  Irrigation changed to ‘Irrigation {US}\| market for \| Alloc Def, U’ |
| Green Bell Pepper | Green Bell Pepper {GLO}\| production \| Alloc Def, U | None |
| Cabbage red {US} | Cabbage red {GLO}\| production \| Alloc Def, U | Electricity changed to ‘Electricity, low voltage {US}\| market group for \| Alloc Def, U’  Irrigation changed to ‘Irrigation {US}\| market for \| Alloc Def, U’ |
| Celery {US} | Celery {GLO}\| 675 production \| Alloc Def, U | Electricity changed to ‘Electricity, low voltage {US}\| market group for \| Alloc Def, U’  Irrigation changed to ‘Irrigation {US}\| market for \| Alloc Def, U’ |
| Cucumbers {US} | Cucumber {GLO}\| production \| Alloc Def, U | Electricity changed to ‘Electricity, low voltage {US}\| market group for \| Alloc Def, U’  Irrigation changed to ‘Irrigation {US}\| market for \| Alloc Def, U’ |
| Aubergine {US} | Aubergine {GLO}\| production \| Alloc Def, U | Electricity changed to ‘Electricity, low voltage {US}\| market group for \| Alloc Def, U’  Irrigation changed to ‘Irrigation {US}\| market for \| Alloc Def, U’ |
| Onion {US} | Onion {GLO}\| 855 production \| Alloc Def, U | Electricity changed to ‘Electricity, low voltage {US}\| market group for \| Alloc Def, U’  Irrigation changed to ‘Irrigation {US}\| market for \| Alloc Def, U’ |
| Fava bean, organic {GLO} | Fava bean, organic {GLO}\| Market for \| Alloc Def, U | Transport to market removed |
| Avocado {GLO} | Avocado {GLO}\| production \| Alloc Def, U | None |
| Carrot {US} | Carrot {GLO}\| production \| Alloc Def, U | Electricity changed to ‘Electricity, low voltage {US}\| market group for \| Alloc Def, U’  Irrigation changed to ‘Irrigation {US}\| market for \| Alloc Def, U’ |
| Tomato {US} | Tomato {GLO}\| production \| Alloc Def, U | Electricity changed to ‘Electricity, low voltage {US}\| market group for \| Alloc Def, U’  Irrigation changed to ‘Irrigation {US}\| market for \| Alloc Def, U’ |
| Potato {US} | Potato {US}\| production \| Alloc Def, U | None |
| Citrus {US} | Citrus {GLO}\| production \| Alloc Def, U | Electricity changed to ‘Electricity, low voltage {US}\| market group for \| Alloc Def, U’  Irrigation changed to ‘Irrigation {US}\| market for \| Alloc Def, U’ |
| Strawberry {US} | Strawberry {GLO}\| production \| Alloc Def, U | Electricity changed to ‘Electricity, low voltage {US}\| market group for \| Alloc Def, U’  Irrigation changed to ‘Irrigation {US}\| market for \| Alloc Def, U’ |
| Melon {US} | Melon {GLO}\| production \| Alloc Def, U | Electricity changed to ‘Electricity, low voltage {US}\| market group for \| Alloc Def, U’  Irrigation changed to ‘Irrigation {US}\| market for \| Alloc Def, U’ |
| Apple {US} | Apple {GLO}\| production \| Alloc Def, U | Electricity changed to ‘Electricity, low voltage {US}\| market group for \| Alloc Def, U’  Irrigation changed to ‘Irrigation {US}\| market for \| Alloc Def, U’ |
| Banana {GLO} | Banana {GLO}\| production \| Alloc Def, U | None |
| Grape {US} | Grape {GLO}\| production \| Alloc Def, U | Electricity changed to ‘Electricity, low voltage {US}\| market group for \| Alloc Def, U’  Irrigation changed to ‘Irrigation {US}\| market for \| Alloc Def, U’ |
| Pear {US} | Pear {GLO}\| production \| Alloc Def, U | Electricity changed to ‘Electricity, low voltage {US}\| market group for \| Alloc Def, U’  Irrigation changed to ‘Irrigation {US}\| market for \| Alloc Def, U’ |
| Cheese {US} | Cheese, from cow milk, fresh, unripened {GLO}\| cheese production, soft, from cow milk \| Alloc Def, U | Electricity changed to ‘Electricity, medium voltage {US}\| market group for \| Alloc Def, U’  Cream and milk changed to US production scenarios (see below) |
| Milk {US} | Cow milk {CA-QC}\| milk production, from cow \| Alloc Def, U | Soybean feed changed to ‘Soybean, feed {GLO}\| production \| Alloc Def, U’  Maize changed to ‘Maize grain, feed {US}\| production \| Alloc Def, U’  1% of impacts allocated to by-product beef production (see below) |
| Yoghurt {US} | Yogurt, from cow milk {CA-QC}\| production \| Alloc Def, U | Milk changed to ‘Milk {US}’  Electricity changed to ‘Electricity, medium voltage {US}\| market group for \| Alloc Def, U’ |
| Soybean Beverage {US} | Soybean beverage {CA-QC}\| production \| Alloc Def, U | Electricity changed to ‘Electricity, medium voltage {US}\| market group for \| Alloc Def, U’ |
| Tofu {US} | Tofu {CA-QC}\| production \| Alloc Def, U | Electricity changed to ‘Electricity, medium voltage {US}\| market group for \| Alloc Def, U’ |
| Palm oil, refined {GLO} | Palm oil, refined {GLO}\| market for \| Alloc Def, S | None |
| Cream {US} | Cream, from cow milk {CA-QC}\| yogurt production, from cow milk \| Alloc Def, U | Electricity changed to ‘Electricity, medium voltage {US}\| market group for \| Alloc Def, U’ |
| Sugar, from sugarcane {GLO} | Sugar, from sugarcane {GLO}\| production \| Alloc Def, U | None |

- 1. **Literature based**

The following sections detail the life cycle inventories built from previous LCAs.

- - 1. Frozen Broccoli

| **Output to Market** | | |
| --- | --- | --- |
| Item | Amount | Unit |
| Frozen Broccoli {US} | 1 | kg |
| **Production Factors** | | |
| Item | Amount | Unit |
| Broccoli {US} | 1 | kg |
| Frozen Food Manufacturing | 1.543200617 | 2002 USD |

- - 1. Frozen Spinach

| **Output to Market** | | |
| --- | --- | --- |
| Item | Amount | Unit |
| Frozen Spinach {US} | 1 | kg |
| **Production Factors** | | |
| Item | Amount | Unit |
| Spinach {US} | 1 | kg |
| Frozen Food Manufacturing | 1.543200617 | 2002 USD |

- - 1. Canned Olives ^8^

| **Output to Market** | | |
| --- | --- | --- |
| Item | Amount | Unit |
| Canned Olives {US} | 1 | kg |
| **Production Factors** | | |
| Item | Amount | Unit |
| Pesticide, unspecified {GLO}\| market for \| Alloc Def, U | 0.003333 | kg |
| Irrigation {ES}\| market for \| Alloc Def, U | 1 | L |
| Nitrogen fertiliser, as N {GLO}\| market for \| Alloc Def, U | 0.000145 | kg |
| Diesel {GLO}\| market group for \| Alloc Def, U | 0.008 | kg |
| Lubricating oil {GLO}\| market for \| Alloc Def, U | 0.000475 | kg |
| Fruit and vegetable canning, pickling, and drying | 1.720729275 | 2002 USD |
| **Emissions to Air** | | |
| Substance | Amount | Unit |
| CO_2_ (fossil) | 0.283789 | kg |

- - 1. Canned Mushrooms

Reliable LCI data on mushrooms are lacking. We estimate mushroom impacts as the average of all fresh vegetables, akin to Heller and Keoleian ^9^. We add to the average the impacts from canning, taken as $1.80 2002 USD demand from the ‘Fruit and vegetable canning, pickling, and drying’ sector per kilogram canned mushrooms on the market.

- - 1. Canned Beans

Mass change during production taken from the USDA Food Intakes Converted to Retail Commodities Database (FICRCD) ^10^

| **Output to Market** | | |
| --- | --- | --- |
| Item | Amount | Unit |
| Canned Beans {US} | 1 | kg |
| **Production Factors** | | |
| Item | Amount | Unit |
| Fava bean, organic {GLO} | 1.14 | kg |
| Fruit and vegetable canning, pickling, and drying | 1.720729275 | 2002 USD |

- - 1. Canned Cucumbers

Mass change during production taken from the USDA Food Intakes Converted to Retail Commodities Database (FICRCD) ^10^

| **Output to Market** | | |
| --- | --- | --- |
| Item | Amount | Unit |
| Canned Cucumbers {US} | 1 | kg |
| **Production Factors** | | |
| Item | Amount | Unit |
| Cucumber {US} | 0.916 | kg |
| Fruit and vegetable canning, pickling, and drying | 1.720729275 | 2002 USD |

- - 1. Frozen Beans

| **Output to Market** | | |
| --- | --- | --- |
| Item | Amount | Unit |
| Frozen Beans {US} | 1 | kg |
| **Production Factors** | | |
| Item | Amount | Unit |
| Fava bean, organic {GLO} | 1 | kg |
| Frozen Food Manufacturing | 1.543200617 | 2002 USD |

- - 1. Dried Onions

Mass change during production taken from the USDA Food Intakes Converted to Retail Commodities Database (FICRCD) ^10^

| **Output to Market** | | |
| --- | --- | --- |
| Item | Amount | Unit |
| Dried Onion {US} | 1 | kg |
| **Production Factors** | | |
| Item | Amount | Unit |
| Onion {US} | 9.1 | kg |
| Fruit and vegetable canning, pickling, and drying | 1.720729275 | 2002 USD |

- - 1. Canned Carrots

Mass change during production taken from the USDA Food Intakes Converted to Retail Commodities Database (FICRCD) ^10^

| **Output to Market** | | |
| --- | --- | --- |
| Item | Amount | Unit |
| Canned Carrots {US} | 1 | kg |
| **Production Factors** | | |
| Item | Amount | Unit |
| Carrots {US} | 1.12 | kg |
| Fruit and vegetable canning, pickling, and drying | 1.720729275 | 2002 USD |

- - 1. Canned Peppers

Mass change during production taken from the USDA Food Intakes Converted to Retail Commodities Database (FICRCD) ^10^

| **Output to Market** | | |
| --- | --- | --- |
| Item | Amount | Unit |
| Canned Peppers {US} | 1 | kg |
| **Production Factors** | | |
| Item | Amount | Unit |
| Pepper {US} | 1.04 | kg |
| Fruit and vegetable canning, pickling, and drying | 1.720729275 | 2002 USD |

- - 1. Canned Tomatoes

| **Output to Market** | | |
| --- | --- | --- |
| Item | Amount | Unit |
| Canned Tomatoes {US} | 1 | kg |
| **Production Factors** | | |
| Item | Amount | Unit |
| Tomato {US} | 1 | kg |
| Fruit and vegetable canning, pickling, and drying | 1.720729275 | 2002 USD |

- - 1. Frozen Carrots

| **Output to Market** | | |
| --- | --- | --- |
| Item | Amount | Unit |
| Frozen Carrots {US} | 1 | kg |
| **Production Factors** | | |
| Item | Amount | Unit |
| Carrot {US} | 1 | kg |
| Frozen Food Manufacturing | 1.543200617 | 2002 USD |

- - 1. Canned Corn

Mass change during production taken from the USDA Food Intakes Converted to Retail Commodities Database (FICRCD) ^10^

| **Output to Market** | | |
| --- | --- | --- |
| Item | Amount | Unit |
| Canned Corn {US} | 1 | kg |
| **Production Factors** | | |
| Item | Amount | Unit |
| Maize grain {US}\| production \| Alloc Def, U | 0.79 | kg |
| Fruit and vegetable canning, pickling, and drying | 1.720729275 | 2002 USD |

- - 1. Frozen Corn

| **Output to Market** | | |
| --- | --- | --- |
| Item | Amount | Unit |
| Frozen Corn {US} | 1 | kg |
| **Production Factors** | | |
| Item | Amount | Unit |
| Maize grain {US}\| production \| Alloc Def, U | 1 | kg |
| Frozen Food Manufacturing | 1.543200617 | 2002 USD |

- - 1. Frozen Potatoes

| **Output to Market** | | |
| --- | --- | --- |
| Item | Amount | Unit |
| Frozen Potatoes {US} | 1 | kg |
| **Production Factors** | | |
| Item | Amount | Unit |
| Potato {US}\| production \| Alloc Def, U | 1 | kg |
| Frozen Food Manufacturing | 1.543200617 | 2002 USD |

- - 1. Dried Potatoes

Mass change during production taken from the USDA Food Intakes Converted to Retail Commodities Database (FICRCD) ^10^

| **Output to Market** | | |
| --- | --- | --- |
| Item | Amount | Unit |
| Dried Potatoes {US} | 1 | kg |
| **Production Factors** | | |
| Item | Amount | Unit |
| Potato {US}\| production \| Alloc Def, U | 4.819277108 | kg |
| Fruit and vegetable canning, pickling, and drying | 1.720729275 | 2002 USD |

- - 1. Orange Juice

Mass change during production taken from the USDA Food Intakes Converted to Retail Commodities Database (FICRCD) ^10^

| **Output to Market** | | |
| --- | --- | --- |
| Item | Amount | Unit |
| Orange Juice {US} | 1 | kg |
| **Production Factors** | | |
| Item | Amount | Unit |
| Citrus {US} | 2 | kg |

- - 1. Grapefruit Juice

Mass change during production taken from the USDA Food Intakes Converted to Retail Commodities Database (FICRCD) ^10^

| **Output to Market** | | |
| --- | --- | --- |
| Item | Amount | Unit |
| Grapefruit Juice {US} | 1 | kg |
| **Production Factors** | | |
| Item | Amount | Unit |
| Citrus {US} | 2.25 | kg |

- - 1. Apple Juice

Mass change during production taken from the USDA Food Intakes Converted to Retail Commodities Database (FICRCD) ^10^

| **Output to Market** | | |
| --- | --- | --- |
| Item | Amount | Unit |
| Apple Juice {US} | 1 | kg |
| **Production Factors** | | |
| Item | Amount | Unit |
| Apple {US} | 1.5 | kg |

- - 1. Cranberry Juice

Mass change during production taken from the USDA Food Intakes Converted to Retail Commodities Database (FICRCD) ^10^

| **Output to Market** | | |
| --- | --- | --- |
| Item | Amount | Unit |
| Cranberry Juice {US} | 1 | kg |
| **Production Factors** | | |
| Item | Amount | Unit |
| Strawberry {US}* | 1.3 | kg |

* Strawberry taken as surrogate for cranberry. Strawberry {US} process adapted for energy and irrigation in same manner as shown in table 2

- - 1. Grape Juice

Mass change during production taken from the USDA Food Intakes Converted to Retail Commodities Database (FICRCD) ^10^

| **Output to Market** | | |
| --- | --- | --- |
| Item | Amount | Unit |
| Grape Juice {US} | 1 | kg |
| **Production Factors** | | |
| Item | Amount | Unit |
| Grape {US}* | 1.3 | kg |

* Grape {US} process adapted for energy and irrigation in same manner as shown in table 2

- - 1. Pineapple Juice

Mass change during production taken from the USDA Food Intakes Converted to Retail Commodities Database (FICRCD) ^10^

| **Output to Market** | | |
| --- | --- | --- |
| Item | Amount | Unit |
| Pineapple Juice {US} | 1 | kg |
| **Production Factors** | | |
| Item | Amount | Unit |
| Pineapple {GLO}\| production \| Alloc Def, U | 1.88 | kg |

- - 1. Canned Apples

Mass change during production taken from the USDA Food Intakes Converted to Retail Commodities Database (FICRCD) ^10^

| **Output to Market** | | |
| --- | --- | --- |
| Item | Amount | Unit |
| Canned Apples {US} | 1 | kg |
| **Production Factors** | | |
| Item | Amount | Unit |
| Apple {US} | 1.018 | kg |
| Fruit and vegetable canning, pickling, and drying | 1.720729275 |  |

- - 1. Raisins

Mass change during production taken from the USDA Food Intakes Converted to Retail Commodities Database (FICRCD) ^10^

| **Output to Market** | | |
| --- | --- | --- |
| Item | Amount | Unit |
| Raisins {US} | 1 | kg |
| **Production Factors** | | |
| Item | Amount | Unit |
| Grape {US} | 4.52 | kg |
| Fruit and vegetable canning, pickling, and drying | 1.720729275 | 2002 USD |

- - 1. Wheat Flour

| **Output to Market** | | |
| --- | --- | --- |
| Item | Amount | Unit |
| Wheat Flour {US} | 1 | kg |
| **Production Factors** | | |
| Item | Amount | Unit |
| Wheat grain {US}\| wheat production \| Alloc Def, U | 1 | kg |
| Flour milling and malt manufacturing | 0.32137825 | 2002 USD |

- - 1. Processed Rice

| **Output to Market** | | |
| --- | --- | --- |
| Item | Amount | Unit |
| Processed Rice {US} | 1 | kg |
| **Production Factors** | | |
| Item | Amount | Unit |
| Maize grain {US}\| production \| Alloc Def, U | 1 | kg |
| Wet corn milling | 0.423391068 | 2002 USD |

- - 1. Oat Products

| **Output to Market** | | |
| --- | --- | --- |
| Item | Amount | Unit |
| Oat Products {US} | 1 | kg |
| **Production Factors** | | |
| Item | Amount | Unit |
| Oat grain {CA-QC}\| oat production \| Alloc Def, U | 1 | kg |
| Flour milling and malt manufacturing | 0.32137825 | 2002 USD |

- - 1. Ice Cream

| **Output to Market** | | |
| --- | --- | --- |
| Item | Amount | Unit |
| Ice Cream {US} | 1 | kg |
| **Production Factors** | | |
| Item | Amount | Unit |
| Cream {US} | 1 | kg |
| Ice cream and frozen dessert manufacturing | 1.263437634 | 2002 USD |

- - 1. Condensed Milk

Mass change during production taken from the USDA Food Intakes Converted to Retail Commodities Database (FICRCD) ^10^

| **Output to Market** | | |
| --- | --- | --- |
| Item | Amount | Unit |
| Condensed Milk {US} | 1 | kg |
| **Production Factors** | | |
| Item | Amount | Unit |
| Milk {US} | 1.23 | kg |
| Evaporation of milk {US}\| milk evaporation \| Alloc Def, U* | 1.23 | kg |

* Energy and heating sources changed to US

- - 1. Eggs ^11^

| **Output to Market** | | |
| --- | --- | --- |
| Item | Amount | Unit |
| Layer Feed | 1 | kg |
| **Production Factors** | | |
| Item | Amount | Unit |
| Maize grain {GLO}\| market for \| Alloc Def, U | 0.712555066 | kg |
| Soybean meal {GLO}\| market for \| Alloc Def, U | 0.212555066 | kg |
| Lime {GLO}\| market for \| Alloc Def, U | 0.074889868 | kg |

| **Output to Market** | | |
| --- | --- | --- |
| Item | Amount | Unit |
| Pullet | 1 | pullet |
| **Production Factors** | | |
| Item | Amount | Unit |
| Layer Feed | 5.27 | kg |
| Irrigation {US}\| market for \| Alloc Def, U | 9.22 | kg |
| Electricity, low voltage {US}\| market group for \| Alloc Def, U | 3.015 | MJ |
| Diesel, low-sulfur {GLO}\| market group for \| Alloc Def, U | 0.00246 | kg |
| Petrol, unleaded {RoW}\| market for \| Alloc Def, U | 0.0022 | kg |
| Propane {GLO}\| market for \| Alloc Def, U | 0.0357 | kg |
| Natural gas, high pressure {US}\| market for \| Alloc Def, U | 0.180 | ft^3^ |
| **Emissions to Air** | | |
| Substance | Amount | Unit |
| Carbon dioxide (fossil) | 0.61734 | kg |

| **Output to Market** | | |
| --- | --- | --- |
| Item | Amount | Unit |
| Eggs {US} | 1 | kg |
| **By-products** | | |
| Item | Amount | Unit |
| Chicken for slaughtering, live weight {GLO}\| chicken production \| Alloc Def, U | 0.02 | kg |
| **Production Factors** | | |
| Item | Amount | Unit |
| Pullet | 0.036 | pullets |
| Layer Feed | 1.980 | kg |
| Irrigation {US}\| market for \| Alloc Def, U | 4.26 | kg |
| Electricity, low voltage {US}\| market group for \| Alloc Def, U | 0.557 | MJ |
| Diesel, low-sulfur {GLO}\| market group for \| Alloc Def, U | 0.00162 | kg |
| Petrol, unleaded {RoW}\| market for \| Alloc Def, U | 0.000207 | kg |
| Propane {GLO}\| market for \| Alloc Def, U | 0.00175 | kg |
| Natural gas, high pressure {US}\| market for \| Alloc Def, U | 0.00386 | ft^3^ |
| Poultry processing | 1.920739745 | 2002 USD |
|  |  |  |
| **Emissions to Air** | | |
| Substance | Amount | Unit |
| Carbon dioxide (fossil) | 0.02084 | kg |

- - 1. Dried Beans

| **Output to Market** | | |
| --- | --- | --- |
| Item | Amount | Unit |
| Dried Beans {US} | 1 | kg |
| **Production Factors** | | |
| Item | Amount | Unit |
| Fava bean, organic {GLO} | 1.13* | kg |
| Fruit and vegetable canning, pickling, and drying | 1.720729275 | 2002 USD |

* Adjusted for 11.33% water content from USDA for raw pinto beans (item 16042): <https://ndb.nal.usda.gov/ndb/search/list>

- - 1. Beef, feedlot finished ^12^

The LCI is provided for one kilogram of live weight beef for slaughter, which is then adjusted for the carcass yield (the ratio of meat to live weight). An upper carcass yield of 0.428 was taken from the Cornell Small Farms Program ^13^. A lower carcass yield of 0.390 was taken as the product of the dressing percentage of 0.59 (11902714932 kg carcass weight produced/34407300 head slaughtered/580 kg live weight per head in 2011) ^14^ and carcass cutting yield of 0.65 (24.6 kg beef on market/person * 314100000 people/11902714932 kg carcass weight produced in 2011) ^3^.

| **Output to Market** | | |
| --- | --- | --- |
| Item | Amount | Unit |
| Beef, feedlot finished {US} | yield | kg |
| **By-products** | | |
| Item | Amount | Unit |
| Liquid manure spreading, by vacuum tanker {GLO}\| market for \| Alloc Def, U | 0.0041 | m^3^ |
| **Production Factors** | | |
| Item | Amount | Unit |
| Natural gas, high pressure {CA-AB}\| natural gas production \| Alloc Def, U | 0.798 | m^3^ |
| Diesel {GLO}\| market group for \| Alloc Def, U | 0.00303 | kg |
| Electricity, low voltage {MRO, US only}\| market for \| Alloc Def, U | 0.08067 | kWh |
| Alfalfa-grass silage {GLO}\| market for \| Alloc Def, U | 0.431 | kg |
| Maize silage {GLO}\| market for \| Alloc Def, U | 1.04 | kg |
| Maize grain, feed {GLO}\| market for \| Alloc Def, U | 3.49 | kg |
| Energy feed, gross {GLO}\| soybean meal to generic market for protein feed \| Alloc Def, U | 0.00676 | MJ |
| Irrigation {US}\| market for \| Alloc Def, U* | 0.152 | m^3^ |
| **Emissions to Air** | | |
| Substance | Amount | Unit |
| Carbon dioxide (fossil) | 1.56957 | kg |
| Methane | 0.238468168 | kg |
| Ammonia | 0.0193 | kg |
| Nitrate | 0.248 | kg |

* Blue water taken from Mekonnen and Hoekstra, 2012 ^15^

- - 1. Beef, backgrounding-feedlot finished ^12^

Yield calculated in the same manner as above.

| **Output to Market** | | |
| --- | --- | --- |
| Item | Amount | Unit |
| Beef, backgrounding-feedlot finished {US} | yield | kg |
| **By-products** | | |
| Item | Amount | Unit |
| Liquid manure spreading, by vacuum tanker {GLO}\| market for \| Alloc Def, U | 0.0041 | m^3^ |
| **Production Factors** | | |
| Item | Amount | Unit |
| Occupation, pasture and meadow* | 85.7 | m^2^a |
| Natural gas, high pressure {CA-AB}\| natural gas production \| Alloc Def, U | 0.798 | m^3^ |
| Diesel {GLO}\| market group for \| Alloc Def, U | 0.00303 | kg |
| Electricity, low voltage {MRO, US only}\| market for \| Alloc Def, U | 0.08067 | kWh |
| Alfalfa-grass silage {GLO}\| market for \| Alloc Def, U | 0.364 | kg |
| Maize silage {GLO}\| market for \| Alloc Def, U | 3.43 | kg |
| Wheat Pasture {US-midwest} | 12.5 | kg |
| Irrigation {US}\| market for \| Alloc Def, U** | 0.234 | m^3^ |
| **Emissions to Air** | | |
| Substance | Amount | Unit |
| Carbon dioxide (fossil) | 1.56957 | kg |
| Methane | 0.238468168 | kg |
| Ammonia | 0.0193 | kg |
| Nitrate | 0.248 | kg |

* Land occupation taken as the ratio of total land for beef/veal/lamb pasture from Table 8 of the USDA 2012 Census of Agriculture (1.68 * 10^12^ m^2^) ^16^ divided by total market availability of red meat in live weight for that year (1.9*10^10^ kg)^14^

** Blue water for taken from Mekonnen and Hoekstra, 2012 ^15^

- - 1. Beef, culled dairy cattle

Beef and milk are co-products in this system. Mass based allocation is used to apportion the impacts to each product. Average US dairy cow has 2.63 years of milk production ^17^, producing on average 9566 kg milk/annum in the U.S. ^18^ and a slaughter weight of 566 kg for the top three dairy breeds ^19^, then given the two carcass yields of 0.390 and 0.428 there is either 0.0087 kg beef at market/kg milk or 0.0095 kg beef at market/kg milk produced, respectively. Impacts from the milk production system are thus allocated accordingly. Alternatively an economic allocation of $600 ^17^ for the slaughtered dairy cow and 0.531 USD/kg milk ^20^ would yield an allocation of 4.3% of milk impacts to the beef by-product, which is relatively significant, but not critical to the results here (~1% decrease in results for MUD).

- - 1. Ground beef

Beef for ground beef comes from two streams, dedicated beef herds (split 50/50 between feedlot and backgrounding-feedlot finished ^12^) and culled dairy cattle. We assume that all culled dairy cattle go to ground beef due to the low quality of the meat. Assuming a steady state cow population of 9252272 dairy cows in 2012 ^16^ and an average life of 4.8 years ^17^, implies 1927557 culled dairy cattle per year. At 566 kg/head and the aforementioned carcass yields, between 425488932 and 466946828 kg of ground beef from dairy cattle are hitting the market annually.

Of the 7781113636 kg of total beef on the U.S market in 2012, it is estimated by industry that 50% of this was ground beef ^21^. We also used the Center for Disease Control and Preventions National Nutrition and Health Examination Survey (NHANES) from 2011/2012 to estimate the amount of ground beef for all beef consumers ^22^. 8-digit USDA food codes were used to disaggregate total mass of food consumed into constituent masses using the FICRCD ^10^. Those food codes that corresponded to ground beef as the main ingredient were added to the ground beef total. A text search was also performed of the meal descriptions to capture any meals that contain ground-beef as a secondary ingredient, with the amount of beef in meals meeting the search criteria added to the ground beef total. The total amount of ground beef was then divided by the total beef amount to come to an estimate of 30% ground beef as percentage of total beef consumption. The difference between our estimate and the industry estimate could come from the underreporting of unhealthy foods in self-reporting dietary surveys or due to the difficulty of capturing all meals containing ground beef as second ingredients in the USDA food descriptions.

Upper and lower bounds for total ground beef on the market are thus 3890556818 and 2334334091 kg/annum, respectively. At a carcass yield of 0.390, between 10.9% and 18.2% of ground beef in the US originates from the dairy herd. At a carcass yield of 0.428, between 12.0% and 20.0% of US ground beef originates from the dairy herd.

| **Output to Market** | | |
| --- | --- | --- |
| Item | Amount | Unit |
| Ground beef {US} | 1 | kg |
| **Production Factors** | | |
| Item | Amount | Unit |
| Beef, feedlot finished {US} | (1-% dairy)/2* | kg |
| Beef, backgrounding-feedlot finished {US} | (1-% dairy)/2* | kg |
| Beef, culled dairy cattle {US} | % dairy | kg |
| Animal (except poultry) slaughtering, rendering, and processing | 4.150212451 | 2002 USD |

* Divide by two to account for the 50/50 split between supplying streams

- - 1. Beef {US}

Taken as 50/50 split between feedlot and backgrounding-feedlot finished, as these are the two dominant production systems in the US ^12^.

| **Output to Market** | | |
| --- | --- | --- |
| Item | Amount | Unit |
| Beef {US} | 1 | kg |
| **Production Factors** | | |
| Item | Amount | Unit |
| Beef, backgrounding-feedlot finished {US} | 1 | kg |
| Beef, culled dairy cattle {US} | 1 | kg |
| Animal (except poultry) slaughtering, rendering, and processing | 4.150212451 | 2002 USD |

- - 1. Pork ^23^

Austrian pork production used as surrogate for US production.

| **Output to Market** | | |
| --- | --- | --- |
| Item | Amount | Unit |
| Pork {US} | 1 | kg |
| **By-products** | | |
| Item | Amount | Unit |
| Nitrogen fertiliser, as N {GLO}\| field application of ammonium chloride \| Alloc Def, U | 0.030176 | kg |
| Phosphate fertiliser, as P2O5 {GLO}\| market for \| Alloc Def, U | 0.010938 | kg |
| Potassium chloride, as K2O {GLO}\| market for \| Alloc Def, U | 0.027637 | kg |
| **Production Factors** | | |
| Item | Amount | Unit |
| Maize grain {GLO}\| market for \| Alloc Def, U | 1.484375 | kg |
| Wheat grain, feed {GLO}\| market for \| Alloc Def, U | 0.742188 | kg |
| Barley grain, feed {GLO}\| market for \| Alloc Def, U | 0.742188 | kg |
| Protein feed, 100% crude {GLO}\| soybean meal to generic market for protein feed \| Alloc Def, U | 0.283203 | kg |
| Protein feed, 100% crude {GLO}\| rape meal to generic market for protein feed \| Alloc Def, U | 0.146484 | kg |
| Sunflower silage {RoW}\| catch crop growing, sunflower, August-October, organic fertiliser 20 kg N \| Alloc Def, U | 0.146484 | kg |
| Irrigation {US}\| market for \| Alloc Def, U | 0.011738 | m^3^ |
| Electricity, low voltage {US}\| market group for \| Alloc Def, U | 0.341797 | kWh |
| Heat, central or small-scale, other than natural gas {GLO}\| market group for \| Alloc Def, U | 0.185547 | kWh |
| Animal (except poultry) slaughtering, rendering, and processing | 4.150212451 | 2002 USD |
| **Emissions to Air** | | |
| Substance | Amount | Unit |
| Methane | 0.032353516 | kg |
| Ammonia | 6.84E-05 | kg |
| Nitrous Oxide | 0.015615 | kg |

- - 1. Chicken

| **Output to Market** | | |
| --- | --- | --- |
| Item | Amount | Unit |
| Chicken {US} | 1 | kg |
| **Production Factors** | | |
| Item | Amount | Unit |
| Chicken for slaughtering, live weight {GLO}\| chicken production \| Alloc Def, U | 1.33* | kg |
| Poultry processing | 1.920739745 | 2002 USD |

* Carcass yield taken from ^13^

- - 1. Peanuts ^24^

| **Output to Market** | | |
| --- | --- | --- |
| Item | Amount | Unit |
| Peanuts {US} | 1 | kg |
| **Production Factors** | | |
| Item | Amount | Unit |
| Occupation, agriculture | 5.88 | m^2^a |
| Rye seed, for sowing {GLO}\| market for \| Alloc Def, U | 0.005317073 | kg |
| Lime {GLO}\| market for \| Alloc Def, U | 0.317195122 | kg |
| Diesel, low-sulfur {GLO}\| market group for \| Alloc Def, U | 0.044927 | kg |
| Borax, anhydrous, powder {GLO}\| market for \| Alloc Def, U | 0.000170732 | kg |
| Pesticide, unspecified {GLO}\| market for \| Alloc Def, U | 0.007634146 | kg |
| Electricity, low voltage {US}\| market group for \| Alloc Def, U | 0.062317 | kWh |
| Irrigation {US}\| market for \| Alloc Def, U | 0.246341 | m^3^ |
| **Emissions to Air** | | |
| Substance | Amount | Unit |
| Carbon dioxide (fossil) | 0.141951 | kg |

- - 1. Almonds ^25^

| **Output to Market** | | |
| --- | --- | --- |
| Item | Amount | Unit |
| Almonds {US} | 1 | kg |
| **Production Factors** | | |
| Item | Amount | Unit |
| Occupation, agriculture | 0.445 | m^2^a |
| Petrol, unleaded {RoW}\| market for \| Alloc Def, U | 0.0297 | kg |
| Diesel, low-sulfur {GLO}\| market group for \| Alloc Def, U | 0.0398 | kg |
| 2,4-dichlorophenol {GLO}\| market for \| Alloc Def, U | 0.000145 | kg |
| Sulfur {GLO}\| market for \| Alloc Def, U | 0.034 | kg |
| Nitrogen fertiliser, as N {GLO}\| market for \| Alloc Def, U | 0.0609 | kg |
| Zinc {GLO}\| market for \| Alloc Def, U | 0.00125 | kg |
| Irrigation {US}\| market for \| Alloc Def, U | 4.98 | m^3^ |
| Electricity, low voltage {WECC, US only}\| market for \| Alloc Def, U | 1.5 | kWh |
| **Emissions to Air** | | |
| Substance | Amount | Unit |
| Carbon dioxide (fossil) | 0.1557 | kg |

- - 1. Walnuts ^25^

| **Output to Market** | | |
| --- | --- | --- |
| Item | Amount | Unit |
| Walnuts {US} | 1 | kg |
| **Production Factors** | | |
| Item | Amount | Unit |
| Occupation, agriculture | 0.178 | m^2^a |
| Petrol, unleaded {RoW}\| market for \| Alloc Def, U | 0.0162 | kg |
| Diesel, low-sulfur {GLO}\| market group for \| Alloc Def, U | 0.0078 | kg |
| 2,4-dichlorophenol {GLO}\| market for \| Alloc Def, U | 0.000284 | kg |
| Nitrogen fertiliser, as N {GLO}\| market for \| Alloc Def, U | 0.0183 | kg |
| Irrigation {US}\| market for \| Alloc Def, U | 4.98 | m^3^ |
| Electricity, low voltage {WECC, US only}\| market for \| Alloc Def, U | 0.164 | kWh |
| **Emissions to Air** | | |
| Substance | Amount | Unit |
| Carbon dioxide (fossil) | 0.0409 | kg |

- - 1. Cashew ^26^

Austrian pork production used as surrogate for US production.

| **Output to Market** | | |
| --- | --- | --- |
| Item | Amount | Unit |
| Cashew Nuts {BR} | 1 | kg |
| **By-products** | | |
| Item | Amount | Unit |
| See source* | 1.27 | kg |
| **Production Factors** | | |
| Item | Amount | Unit |
| Arable land use, soy bean, Brazil | 0.0577 | m^2^a |
| Limestone, crushed, washed {GLO}\| market for \| Alloc Def, U | 0.63473745 | kg |
| Gypsum, mineral {GLO}\| market for \| Alloc Def, U | 0.028851702 | kg |
| Copper {GLO}\| market for \| Alloc Def, U | 9.60185E-6 | kg |
| Manganese {GLO}\| market for \| Alloc Def, U | 2.40046E-5 | kg |
| Molybdenum {GLO}\| market for \| Alloc Def, U | 1.20023E-6 | kg |
| Zinc {GLO}\| market for \| Alloc Def, U | 0.000108021 | kg |
| Iron pellet {GLO}\| market for \| Alloc Def, U | 3.60069E-5 | kg |
| Urea, as N {GLO}\| market for \| Alloc Def, U | 0.203084247 | kg |
| Phosphate fertiliser, as P2O5 {RoW}\| single superphosphate production \| Alloc Def, U | 0.465089152 | kg |
| Potassium chloride, as K2O {GLO}\| market for \| Alloc Def, U | 0.054010387 | kg |
| Glyphosate {GLO}\| market for \| Alloc Def, U | 0.001436815 | kg |
| Diesel {GLO}\| market group for \| Alloc Def, U | 0.089118725 | kg |
| Irrigation {BR}\| market for \| Alloc Def, U | 5.476053087 | m^3^ |

* Mass allocation performed

- - 1. Fresh and frozen fish/Canned Fish/Canned Sardines

| **Output to Market** | | |
| --- | --- | --- |
| Item | Amount | Unit |
| Salmon, Atlantic {ES} | 1 | kg |
| **Production Factors** | | |
| Item | Amount | Unit |
| Freight ship, transoceanic {GLO}\| market for \| Alloc Def, U | 7.10997E-09 | ship |
| Diesel, low-sulfur {GLO}\| market group for \| Alloc Def, U | 0.359335038 | kg |
| Alkyd paint, white, without water, in 60% solution state {GLO}\| market for \| Alloc Def, U | 8.95141E-05 | kg |
| **Emissions to Air** | | |
| Substance | Amount | Unit |
| Carbon dioxide (fossil) | 1.135549872 | kg |

Taken from Hospido et al., 2005 ^27^

| **Output to Market** | | |
| --- | --- | --- |
| Item | Amount | Unit |
| Fish feed | 1 | kg |
| **Production Factors** | | |
| Item | Amount | Unit |
| Rape seed, organic {CH}\| production \| Alloc Def, U | 0.061 | m^2^a |
| Rape oil, crude {CH}\| market for \| Alloc Def, U | 0.041 | kg |
| Rape meal {GLO}\| to generic market for protein feed \| Conseq, U | 0.051 | kg |
| Protein pea {GLO}\| market for \| Alloc Def, U, U | 0.051 | kg |
| Maize grain, feed {GLO}\| market for \| Alloc Def, U | 0.092 | kg |
| Energy feed, gross {GLO}\| skimmed milk, from cow milk to generic market for protein feed \| Alloc Rec, U | 0.056 | MJ |
| Wheat grain, feed {GLO}\| market for \| Alloc Def, U | 0.133 | kg |
| **Emissions to Air** | | |
| Substance | Amount | Unit |
| Carbon dioxide (fossil) | 1.2848 | kg |
| Sulfur dioxide | 0.0076 | kg |

Taken from Pelletier et al., 2009 ^28^

| **Output to Market** | | |
| --- | --- | --- |
| Item | Amount | Unit |
| Salmon, farmed {US} | 1 | kg |
| **Production Factors** | | |
| Item | Amount | Unit |
| Fish feed | 1.313 | m^2^a |
| Transport, freight, lorry >32 metric ton, EURO6 {GLO}\| market for \| Alloc Def, U | 0.3192 | tkm |
| Electricity, high voltage {MRO, US only}\| market for \| Alloc Def, U | 2326.9 | MJ |

Taken from Pelletier et al., 2009 ^28^

| **Output to Market** | | |
| --- | --- | --- |
| Item | Amount | Unit |
| Fresh and frozen fish {US} | 1 | kg |
| **Production Factors** | | |
| Item | Amount | Unit |
| Tuna, Atlantic {ES} | 0.875 | kg |
| Salmon, farmed {US} | 0.125 | kg |
| Seafood product preparation and packaging | 2.274835635 | 2002 USD |

87.5% of fish consumed in US is wild (http://www.seafoodhealthfacts.org/seafood-choices/overview-us-seafood-supply), Atlantic Tuna and farmed Salmon used as proxies for all wild and farmed fish consumed in US respectively

| **Output to Market** | | |
| --- | --- | --- |
| Item | Amount | Unit |
| Canned fish {US} | 1 | kg |
| **Production Factors** | | |
| Item | Amount | Unit |
| Tuna, Atlantic {ES} | 0.865 | kg |
| Salmon, farmed {US} | 0.124 | kg |
| Seafood product preparation and packaging | 2.274835635 | 2002 USD |

87.5% of fish consumed in US is wild (http://www.seafoodhealthfacts.org/seafood-choices/overview-us-seafood-supply), Atlantic Tuna and farmed Salmon used as proxies for all wild and farmed fish consumed in US respectively. Mass change from fresh to canned taken from FICRCD ^10^, the difference being water.

| **Output to Market** | | |
| --- | --- | --- |
| Item | Amount | Unit |
| Canned sardines {US} | 1 | kg |
| **Production Factors** | | |
| Item | Amount | Unit |
| Tuna, Atlantic {ES} | 0.868 | kg |
| Salmon, farmed {US} | 0.124 | kg |
| Seafood product preparation and packaging | 2.274835635 | 2002 USD |

87.5% of fish consumed in US is wild (http://www.seafoodhealthfacts.org/seafood-choices/overview-us-seafood-supply), Atlantic Tuna and farmed Salmon used as proxies for all wild and farmed fish consumed in US respectively. Mass change from fresh to canned taken from FICRCD ^10^, the difference being water.

- - 1. Fresh and frozen shellfish ^29^

| **Output to Market** | | |
| --- | --- | --- |
| Item | Amount | Unit |
| Fresh and frozen shellfish {TH} | 1 | kg |
| **Production Factors** | | |
| Item | Amount | Unit |
| Occupation, water bodies, artificial | 0.411 | m^2^a |
| Fish feed | 1.9 | kg |
| Diesel {GLO}\| market group for \| Alloc Def, U | 0.0397 | kg |
| Liquefied petroleum gas {RoW}\| market for \| Alloc Def, U | 0.08 | kg |
| Seafood product preparation and packaging | 2.274835635 | 2002 USD |
| **Emissions to Air** | | |
| Substance | Amount | Unit |
| Carbon dioxide (fossil) | 0.366 | kg |

* Taken from Pelletier et al., 2009 ^28^

- - 1. Cured fish

| **Output to Market** | | |
| --- | --- | --- |
| Item | Amount | Unit |
| Cured fish {US} | 1 | kg |
| **Production Factors** | | |
| Item | Amount | Unit |
| Tuna, Atlantic {ES} | 2.36 | kg |
| Salmon, farmed {US} | 0.338 | kg |
| Seafood product preparation and packaging | 2.274835635 | 2002 USD |

87.5% of fish consumed in US is wild (http://www.seafoodhealthfacts.org/seafood-choices/overview-us-seafood-supply), Atlantic Tuna and farmed Salmon used as proxies for all wild and farmed fish consumed in US respectively. Mass change from fresh to canned taken from FICRCD ^10^, losses due to dehydration.

- - 1. Plant based burger (PBB)

| **Output to Market** | | |
| --- | --- | --- |
| Item | Amount | Unit |
| PBB Ingredients | 1 | kg |
| **Production Factors** | | |
| Item | Amount | Unit |
| Ammonia, liquid {RER}\| market for \| Alloc Rec, U | 0.0156 | kg |
| Ammonium sulfate, as N {GLO}\| market for \| Alloc Rec, U | 0.00547 | kg |
| Chemical, organic {GLO}\| market for \| Alloc Rec, U | 0.00472096 | kg |
| Boric acid, anhydrous, powder {GLO}\| market for \| Alloc Rec, U | 0.00000005 | kg |
| Calcium Sulfate Dihydrate* | 0.00015 | kg |
| Acetic acid, without water, in 98% solution state {GLO}\| market for \| Alloc Rec, U | 0.13833825 | kg |
| Cobalt {GLO}\| market for \| Alloc Rec, U | 0.00000108 | kg |
| Copper sulfate {GLO}\| market for \| Alloc Rec, U | 0.000013 | kg |
| Iron sulfate {GLO}\| market for \| Alloc Rec, U | 0.00014 | kg |
| Magnesium sulfate {GLO}\| market for \| Alloc Rec, U | 0.00408 | kg |
| Manganese sulfate {GLO}\| market for \| Alloc Rec, U | 0.00000648 | kg |
| Potassium carbonate {GLO}\| market for \| Alloc Rec, U | 0.00328 | kg |
| Sodium hydroxide, without water, in 50% solution state {GLO}\| market for \| Alloc Rec, U | 0.14826 | kg |
| Sodium {GLO}\| market for \| Alloc Rec, U | 0.0000006 | kg |
| Sulfuric acid {GLO}\| market for \| Alloc Rec, U | 0.0000108 | kg |
| Water, completely softened, from decarbonised water, at user {GLO}\| market for \| Alloc Rec, U | 13.991 | kg |
| Zinc {GLO}\| market for \| Alloc Rec, U | 0.0000257 | kg |
| Maize grain {GLO}\| market for \| Alloc Rec, U | 0.4181749 | kg |
| Acetic acid, without water, in 98% solution state {GLO}\| market for \| Alloc Rec, U | 0.13833825 | kg |
| Glycine {GLO}\| market for \| Alloc Rec, U | 0.0169156 | kg |
| Lactic acid {GLO}\| market for \| Alloc Rec, U | 0.00088 | kg |
| Chemical, organic {GLO}\| market for \| Alloc Rec, U | 0.00472096 | kg |
| Sodium hydroxide, without water, in 50% solution state {GLO}\| market for \| Alloc Rec, U | 0.14826 | kg |
| Coconut oil, crude {PH}\| production \| Alloc Rec, U | 0.166 | kg |
| Acetic acid, without water, in 98% solution state {GLO}\| market for \| Alloc Rec, U | 0.13833825 | kg |
| Potato protein* | 0.066 | kg |
| Soybean {GLO}\| market for \| Alloc Rec, U | 0.00209 | kg |
| Wheat gluten meal, consumption mix, at feed compound plant/NL Economic | 0.25 | kg |
| Water, completely softened, from decarbonised water, at user {GLO}\| market for \| Alloc Rec, U | 13.991 | kg |

* Taken from Agri-footprint database

| **Output to Market** | | |
| --- | --- | --- |
| Item | Amount | Unit |
| Electricity, low voltage, US average | 1 | kWh |
| **Production Factors** | | |
| Item | Amount | Unit |
| Electricity, low voltage {ASCC}\| market for \| Alloc Rec, U | 0.002 | kWh |
| Electricity, low voltage {FRCC}\| market for \| Alloc Rec, U | 0.052 | kWh |
| Electricity, low voltage {HICC}\| market for \| Alloc Rec, U | 0.003 | kWh |
| Electricity, low voltage {MRO, US only}\| market for \| Alloc Rec, U | 0.057 | kWh |
| Electricity, low voltage {NPCC, US only}\| market for \| Alloc Rec, U | 0.064 | kWh |
| Electricity, low voltage {RFC}\| market for \| Alloc Rec, U | 0.227 | kWh |
| Electricity, low voltage {SERC}\| market for \| Alloc Rec, U | 0.269 | kWh |
| Electricity, low voltage {SPP}\| market for \| Alloc Rec, U | 0.055 | kWh |
| Electricity, low voltage {TRE}\| market for \| Alloc Rec, U | 0.089 | kWh |
| Electricity, low voltage {WECC, US only}\| market for \| Alloc Rec, U | 0.182 | kWh |

Average US grid mix used since final production location is unknown

| **Output to Market** | | |
| --- | --- | --- |
| Item | Amount | Unit |
| PBB | 1 | kg |
| **Production Factors** | | |
| Item | Amount | Unit |
| PBB Ingredients | 1.1* | kg |
| Electricity, low voltage, US average | 5.165 | kWh |
| Transport, freight, lorry 16-32 metric ton, EURO6 {GLO}\| market for \| Alloc Rec, U | 1.35 | tkm |
| Heat, central or small-scale, natural gas {RoW}\| market for heat, central or small-scale, natural gas \| Alloc Rec, U | 0.161 | kWh |
| Compressed air, 800 kPa gauge {GLO}\| market for \| Alloc Rec, U | 3.22 | m^3^ |
| Carbon dioxide, liquid {RER}\| market for \| Alloc Rec, U | 0.297 | kg |

* 10% loss assumed

- - 1. Vegetable Oil Mix

Taken as margarine from Nilsson et al., 2010 ^30^

| **Output to Market** | | |
| --- | --- | --- |
| Item | Amount | Unit |
| Vegetable Oil Mix {GLO} | 1 | kg |
| **Production Factors** | | |
| Item | Amount | Unit |
| Vegetable oil, refined {GLO}\| palm oil, refined, to generic market for vegetable oil \| Conseq, S | 0.474074074 | kg |
| Soybean oil, refined {GLO}\| to generic market for vegetable oil, refined \| Conseq, U | 0.331851852 | kg |
| Rape oil, crude {Europe without Switzerland}\| rape oil mill operation \| Conseq, U | 0.194074074 | kg |

- - 1. High Fructose Corn Syrup (HFCS)^31^

| **Output to Market** | | |
| --- | --- | --- |
| Item | Amount | Unit |
| HFCS {US} | 1 | kg |
| **By-products** | | |
| Item | Amount | Unit |
| Maize grain, feed {GLO}\| market for \| Alloc Def, U | 0.348 | kg |
| Palm kernel oil, crude {GLO}\| market for \| Alloc Def, U | 0.027 | kg |
| **Production Factors** | | |
| Item | Amount | Unit |
| Maize grain {US}\| production \| Alloc Def, U | 1.5 | kg |
| Lime {GLO}\| market for \| Alloc Def, U | 0.0003 | kg |
| Sulfuric acid {GLO}\| market for \| Alloc Def, U | 0.00045 | kg |
| Sulfur dioxide, liquid {GLO}\| market for \| Conseq, S | 0.00306 | kg |
| Urea, as N {GLO}\| market for \| Alloc Def, U | 0.000208 | kg |
| Sodium chloride, powder {GLO}\| market for \| Alloc Def, U | 0.000065 | kg |
| Sodium hydroxide, without water, in 50% solution state {GLO}\| market for \| Alloc Def, U | 0.000282 | kg |
| Cyclohexane {GLO}\| market for \| Alloc Def, U | 0.000055 | kg |
| Chlorine, liquid {GLO}\| market for \| Alloc Def, U | 0.000012 | kg |
| Water, decarbonised, at user {GLO}\| market for \| Alloc Def, U | 4.9 | kg |
| Electricity, low voltage {US}\| market group for \| Alloc Def, U | 0.934 | MJ |
| Natural gas, high pressure {US}\| market for \| Alloc Def, U | 0.0596 | m^3^ |
| **Emissions to Air** |  |  |
| Substance | Amount | Unit |
| Carbon dioxide (fossil) | 0.17 | kg |

- - 1. Glucose from corn

| **Output to Market** | | |
| --- | --- | --- |
| Item | Amount | Unit |
| Glucose from corn {US} | 1 | kg |
| **Production Factors** | | |
| Item | Amount | Unit |
| Maize grain {US}\| production \| Alloc Def, U | 1.6 | kg |

1. **References**

1. Hendrickson, C. T., Lave, L. B. & Matthews, H. S. *Environmental Life Cycle Assessment of Goods and Services: An Input-Output Approach*. *RFF Press* (2006). doi:10.2307/302397

2. Suh, S. (Ed. . *Handbook of Input-Output Economics in Industrial Ecology*. *Eco-Efficiency in Industry and Science* (2009). doi:10.1007/978-1-4020-5737-3

3. USDA. Food Availability (Per Capita) Data System. (2016). Available at: http://www.ers.usda.gov/data-products/food-availability-(per-capita)-data-system/.aspx. (Accessed: 23rd September 2016)

4. U.S. Department of Commerce - Bureau of Economic Analysis. Benchmark input-output data. (2016). Available at: http://www.bea.gov/industry/io_benchmark.htm. (Accessed: 2nd July 2016)

5. United States Department of Agriculture. Current Agricultural Industrial Reports Program. (2016). Available at: https://www.nass.usda.gov/Surveys/Guide_to_NASS_Surveys/Current_Agricultural_Industrial_Reports/. (Accessed: 27th August 2016)

6. Cortellino, G. in *Handbook of Frozen Food Processing and Packaging, Second Edition* 435–460 (CRC Press, 2011). doi:doi:10.1201/b11204-22

7. Crosson, P. *et al.* A review of whole farm systems models of greenhouse gas emissions from beef and dairy cattle production systems. *Anim. Feed Sci. Technol.* **166–167,** 29–45 (2011).

8. Salomone, R. & Ioppolo, G. Environmental impacts of olive oil production: a Life Cycle Assessment case study in the province of Messina (Sicily).(Case study). *J. Clean. Prod.* **28,** 88 (2012).

9. Heller, M. C. & Keoleian, G. a. Greenhouse Gas Emission Estimates of U.S. Dietary Choices and Food Loss. *J. Ind. Ecol.* **0,** n/a-n/a (2014).

10. USDA. Food Intakes Converted to Retail Commodoties. (2016). Available at: https://www.ars.usda.gov/northeast-area/beltsville-md/beltsville-human-nutrition-research-center/food-surveys-research-group/docs/ficrcd-overview/.

11. Pelletier, N., Ibarburu, M. & Xin, H. Comparison of the environmental footprint of the egg industry in the United States in 1960 and 2010. *Poult. Sci.*  **93,** 241–255 (2014).

12. Pelletier, N., Pirog, R. & Rasmussen, R. Comparative life cycle environmental impacts of three beef production strategies in the Upper Midwestern United States. *Agric. Syst.* **103,** 380–389 (2010).

13. Cornell Small Farms Program. Yields and Dressing Percentages. *Resource Guide to Direct Marketing Livestock and Poultry* (2012). Available at: http://smallfarms.cornell.edu/2012/07/10/yields-and-dressing-percentages/.

14. United States Department of Agriculture - National Agricultural Statistics Services. *Livestock Slaughter 2011 Summary*. (2012).

15. Mekonnen, M. M. & Hoekstra, A. Y. A Global Assessment of the Water Footprint of Farm Animal Products. *Ecosystems* **15,** 401–415 (2012).

16. United States Department of Agriculture. *2012 Census of Agriculture*. (2014).

17. de Vries, A. Cow longevity economics - the cost benefit of keeping the cow in the herd. in *Conference Proceedings of the Cow Longevity Conference* (2013).

18. United States Department of Agriculture - Economic Research Service. *U.S. milk production and related data (quarterly)*. (2016).

19. Holstein Foundation. *Dairy Cattle Reproduction*. (2015).

20. FAO. FAOSTAT - Producer Prices. (2015). Available at: http://faostat3.fao.org/browse/P/PP/E.

21. Beef Checkoff. Total Beef Category Breakdown. (2015). Available at: http://www.beefretail.org/totalbeefcategorybreakdown.aspx.

22. Centers for Disease Control and Prevention. About the National Health and Nutrition Examination Survey. (2015). Available at: http://www.cdc.gov/nchs/nhanes/about_nhanes.htm.

23. Winkler, T., Schopf, K., Aschemann, R. & Winiwarter, W. From farm to fork - A life cycle assessment of fresh Austrian pork. *Journal of Cleaner Production* (2015). doi:10.1016/j.jclepro.2016.01.005

24. University of Arkansas. *National Scan-level Life Cycle Assessment for Production of US Peanut Butter Center for Agricultural and Rural*. (2012).

25. Venkat, K. Comparison of Twelve Organic and Conventional Farming Systems: A Life Cycle Greenhouse Gas Emissions Perspective. *J. Sustain. Agric.* **36,** 620–649 (2012).

26. Brito De Figueirêdo, M. C. *et al.* Environmental assessment of tropical perennial crops: The case of the Brazilian cashew. *J. Clean. Prod.* **112,** 131–140 (2016).

27. Hospido, A. & Tyedmers, P. Life cycle environmental impacts of Spanish tuna fisheries. *Fish. Res.* **76,** 174–186 (2005).

28. Pelletier, N. *et al.* Not all salmon are created equal: Life cycle assessment (LCA) of global salmon farming systems. *Environ. Sci. Technol.* **43,** 8730–8736 (2009).

29. Tantipanatip, W., Jitpukdee, S., Keeratiurai, P., Tantikamton, K. & Thanee, N. Life Cycle Assessment of Pacific White Shrimp (Penaeus vannamei) Farming System in Trang Province, Thailand. *Adv. Mater. Res.* **1030–1032,** 679–682 (2014).

30. Nilsson, K. *et al.* Comparative life cycle assessment of margarine and butter consumed in the UK, Germany and France. *Int. J. Life Cycle Assess.* **15,** 916–926 (2010).

31. Renouf, M. A., Wegener, M. K. & Nielsen, L. K. An environmental life cycle assessment comparing Australian sugarcane with US corn and UK sugar beet as producers of sugars for fermentation. *Biomass and Bioenergy* **32,** 1144–1155 (2008).
